# Effects of Biostimulants on the Chemical Composition of Essential Oil and Hydrosol of Lavandin (*Lavandula x intermedia* Emeric ex Loisel.) Cultivated in Tuscan-Emilian Apennines

**DOI:** 10.3390/molecules26206157

**Published:** 2021-10-12

**Authors:** Eleonora Truzzi, Stefania Benvenuti, Davide Bertelli, Enrico Francia, Domenico Ronga

**Affiliations:** 1 Department of Life Sciences, University of Modena and Reggio Emilia, Via G. Campi 103, 41125 Modena, Italy; eleonora.truzzi@unimore.it (E.T.); davide.bertelli@unimore.it (D.B.); enrico.francia@unimore.it (E.F.); 2 Centre BIOGEST–SITEIA, Department of Life Sciences, University of Modena and Reggio Emilia, Via Amendola 2, 42122 Reggio Emilia, Italy; 3 Pharmacy Department, University of Salerno, Via Giovanni Paolo II 132, 84084 Fisciano, Italy; dronga@unisa.it

**Keywords:** lavandin, biostimulants, essential oil, aromatic plants, chemical composition, *Lavandula*

## Abstract

In recent years, it has been shown that biostimulants can efficiently enhance plant metabolic processes, leading to an increased production of essential oil (EO) in aromatic plants. The present study aimed to evaluate the effects of two different commercial biostimulants composed of amino acids and seaweed extract, normally used for food organic crops, on the production and composition of EO and hydrosol of *Lavandula x intermedia*, cultivar “Grosso”. The products were applied during 2020 growing season on lavender crops in three different locations of the Northern Italian (Emilia-Romagna Region) Apennines. Plants were harvested and EOs extracted by steam distillation and analyzed by gas chromatography. Both biostimulants affected the yield of EO per plant (+11% to +49% depending on the treatment/farm combination) without significantly changing the chemical composition of EOs and hydrosols. Conversely, the composition of EOs and hydrosols are related to the location, and the main compounds of “Grosso” cultivar, limonene, 1,8-cineole, cis-ocimene, linalool, camphor, borneol, terpinen-4-ol, and linalyl acetate, show different ratios at the experimental test sites. The differences might be due to the sunlight exposure and various maintenance of the crops over the years. In conclusion, these results suggest that the employment of biostimulants on lavandin crops do not endanger the quality of the EO while increasing biomass production and promoting the sustainability of the crop.

## 1. Introduction

For decades, essential oils (EOs) have been employed in food, cosmetic, and pharmaceutical industries due to their countless biological activities [1,2,3]. In the last years, the research in aromatic plants expanded towards new applications, as in the agri-food sector for EOs activity to exhibit strong effectiveness against food-borne pathogens and pests [4]. In consideration of the need to limit or exclude antibiotics, pesticides, and preservatives in the food chain, it is important to study and apply new strategies and solutions for bio-sustainable production, conservation, and transformation. For this reason, great efforts at the research level have been made in applying EOs as biopesticides and food bio-preservatives [5,6,7]. Thus, the global market of EOs is destined to grow in the next years and their future application in the agri-food industries must take into account the origin of plant species, the chemical composition, and the relationships between the constituents [8].

EOs are complex mixtures of volatile monoterpenes and sesquiterpenes mainly collected in plant leaves, stems, flowers, fruit peels, and roots. They are secondary metabolites involved mainly in plant defense and adaptation to environmental factors. For most of the aromatic plant species, the yield of EOs is very low, and, in order to handle industrial quantities of plant material, large distillation equipment and considerable consumption of thermal energy are required. For this reason, there are numerous efforts to increase the yield of EOs starting from the application of agricultural interventions, as well as improvements in the extraction technique. In particular, agricultural research is involved in increasing the yield of EO and improving the quality composition [9,10]. In this context, progress has been made in understanding the underlying factors of EO production by plants. In particular, EO biosynthesis was demonstrated to be strictly related to agro-climatic, soil conditions, light exposure, genetic variability, and development stage of the whole plant [11,12]. Moreover, in recent years, the application of plant extracts or biostimulants has been attempted on aromatic plants cultivation [13,14,15,16], also allowed in organic farming. Biostimulants are compounds able to enhance plant nutrition, stress tolerance, and/or plant metabolic processes in order to improve crop quality. Biostimulants can be composed of several ingredients and are generally classified based on their content and source [17]. Amino acids (AA) and peptides are one of the most employed growth promoters. They are obtained from both plant and animal wastes by protein hydrolysis and are widely recognized as promoters of N uptake and precursors of secondary compounds and hormones. Moreover, depending on their structure, AA might be involved in chelation, complexation, or antioxidant processes. In particular, chelating effects are exhibited by proline which protects plants against heavy metal and assists the micronutrients mobility and absorption by roots. Furthermore, the scavenging activity of free radicals is exerted by glycine and betaine, reducing the environmental stress of the plant [18,19]. In the last years, seaweed and microalgae promoter effects have also been recorded [20], even though since ancient times they have been employed as fertilizers. Seaweeds are composed of N-containing compounds, such as glycine and betaine, sterols, and polysaccharides, such as alginates, laminarin, and carrageenans. When directly applied to plants, seaweeds or microalgae act both as nutrients suppliers and stressor protectants. Moreover, besides sterols action, seaweeds can be considered as regulators of hormone biosynthetic genes in plant tissues, inducing a further growth and development of crops [21,22]. Finally, also yeast extracts are receiving an increased acceptance as biostimulants, being rich in vitamins, carbohydrates, nucleic acid, and lipids. Yeast extracts promote the absorption of phosphorous and the production of phytohormones [23,24]. Notwithstanding the well-documented effectiveness, few studies attempted to employ biostimulants on organic *Lavandula* crops to improve their productivity, the yield of EO, and its chemical quality [25,26].

The genus *Lavandula* is one of the most cultivated aromatic plants in Europe, including up to 39 different species. Nowadays, the most common species include *Lavandula angustifolia* Mill. (Lavender), *Lavandula latifolia* Medik. (Broadleaved lavender), and *Lavandula x intermedia* Emeric ex Loisel. (Lavandin). Lavender and lavandin EOs display antimicrobial, antioxidant, and anti-inflammatory activity [27], and in the last years, several studies highlighted their efficacy on pests [28,29,30]. Recently lavender EO was developed and commercialized for its anxiolytic and antidepressive effects, both in humans and animals. All these activities confer to lavender and lavandin EOs a high market versatility and value, making their cultivation a perfect target for agricultural efforts in obtaining a standardized, controlled, and increased EO production.

Hence, the aim of the present study was the evaluation of biostimulant effects on *Lavandula x intermedia* crops in terms of EO yield and composition. Commercial biostimulants rich in AA, yeast, and seaweeds were selected and applied on lavandin crops. The improvements in the cultivation of lavender and lavandin represent a good opportunity for marginal and abandoned lands in the hilly and mountainous belt, such as the Tuscan-Emilian Apennines in the north-center of Italy, and for its resistance to strong thermal changes and diseases. The Apennines were selected as the target region for its optimal pedo-climatic conditions, suitable for aromatic plant crops.

## 2. Results and Discussion

### 2.1. EO Content in Fresh Lavandin Flowers

The fresh lavender flowers and stems were subjected to steam distillation by Clevenger apparatus. The extracted EOs were measured, and the yield percentages were calculated (Table 1). Additionally, the total EO per plant was calculated to infer the effects of the biostimulants on the yield of EOs per plant.

The yields from fresh flowers ranged from 1.04 to 2.80%, depending on the farm. Baydar et al. reported for cultivar (cv.) “Grosso” a yield of 1.00–1.5 % at the same altitude of plant growing [31]. On the contrary, higher yields of about 3% were highlighted by Usano-Alemany et al. from cv. “Grosso” grown under similar pedo-climatic conditions, without either irrigation or fertilization [32].

The results of the statistical analysis on EO content (reported assigning the same latter to groups which were not significantly different) highlighted that the yields of CA EOs were significantly lower than those of PE and PR (*p* < 0.0001). Moreover, the treatments with the biostimulants did not influence the yields of EO within the same farm. On the other hand, by considering the biomass weight per plant, the biostimulants have been observed to significantly increase the EO yield per plant in PR group (+39% and +49% compared to untreated control for T1 and T2, respectively). In the case of CA and PE, a little effect of water and biostimulants treatments on the total yield was observed compared to the CTRL (+26–28% for CA, +28–11% for PE). Similar results were reported by Giannoulis et al., where the application of seaweed-based biostimulant on Lavandula angustifolia crops did not increase the content *w/w* of EOs, but rather the yield per plant compared to the farm control [33]. Thus, biostimulants seemed to increase the total plant biomass rather than increase the content of EO in spikes.

### 2.2. Chemical Composition of the EOs

The fresh-distilled EOs were analyzes by gas chromatography coupled with flame ionization detector (GC-FID) in order to quantify the relative percent abundance of terpenes and identify differences among the treatments and farms (Table 2). Notwithstanding that the EOs were obtained from lavandin crops belonging to three farms with similar agro-climatic (altitude and meteorology) conditions, significant differences in the chemical compositions of the EOs were observed (Table S1). A total of 48 monoterpenes and sesquiterpenes were identified and quantified, but 36 of them were present in all the samples. Noteworthy, the monoterpenes hexenal, α-thujene, p-cymene, trans rose oxide, and geranyl acetate were detected only in the EOs belonging to CA farm, while fenchol, α-copaene, and γ-cadinene were found in PE and PR EOs. Regarding the type of biostimulant treatment, in PE EOs no significant differences in composition were observed, while in PR e CA EOs some terpenes showed a *p* < 0.05 as shown in Table S2. The most important significant differences induced by biostimulants-based treatments were observed in PR EOs. In particular, T2 samples exhibited a higher level of 1,8-cineole and camphor and lower ratios of sabinene, α-phellandrene, borneol, and lavandulol.

The chemical composition of PE and PR EOs resulted in agreement with those reported in the literature by several authors [34,35,36], while the composition of CA EOs turned out to be significantly different. Indeed, the EOs belonging to CA resulted significantly different from the EOs of the other two farms for the percent abundance of 33 terpenes out of a total of 36, with a *p*-value ranging from 0.0001 to 0.019 (Table S1). In particular, CA EO shows monoterpenes hydrocarbons in high quantities in particular limonene and cis-ocimene compare to PE and PR EOs. Alcohols such as linalool, borneol, and terpinen-4-ol are contained in larger quantities in CA EOs, than in PE and PR EOs, while the amounts of their biosynthetic derivatives such as camphor and linalyl acetate are lower.

The composition of the EOs from the different farms was also compared with the International Standards for *L. x intermedia* of cv. “Grosso”, in order to evaluate their compliance with the standard composition. In all the samples, hexyl hexanoate and myrcene resulted out of the ranges. The EOs belonging to PR resulted in compliance with the ISO normative for all the other components, while the relative abundance of all the CA terpenes were out of the standard range. In the case of PE EOs, borneol slightly exceeded the maximum percentage.

In order to summarize the most important differences within the three farms and crop treatments in terms of terpenic composition, principal component analysis (PCA) was performed on the semi-quantitative results by considering the components of the EOs present in all the samples as dependent variables (36 total mono- and sesquiterpenes). PCA is an unsupervised multivariate analysis pattern recognition technique exploited to visually highlight differences among samples and to understand which variables are the most distinguishing of each sample [37].

**Figure 1:**
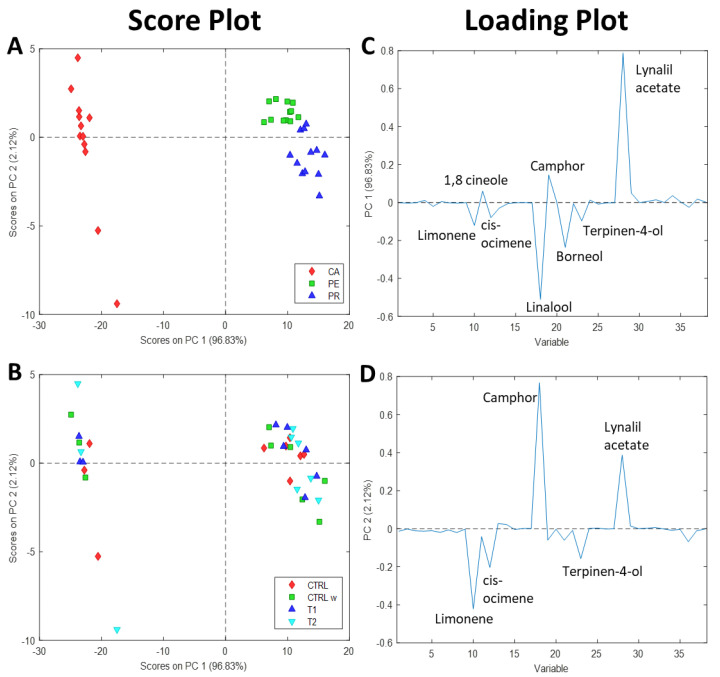
Score plot of principal component analysis (PCA) labeling the farms (**A**) and the treatments (**B**), and loading plots on PC1 (**C**) and PC2 (**D**). CA, Campazzo; PE, Pedroni; PR, Preci; CTRL, farm control; CTRL W, foliar application of water; T1, foliar application of FITOSTIM; T2, foliar application of FITOSTIM ALGA.

The two principal components (PCs) extracted by PCA’s algorithm explained 98.95% of the total variance. In particular, 96.83% and 2.12% of the variance were described by PC1 and PC2, respectively. The high variance described by PC1 might be explained by the fact that the most relevant differences in the dataset were related to the farm origin, and in particular to CA farm, in accordance with ANOVA results (Table S1). As can be seen in the score plot in Figure 1A, the PCs allowed good discrimination of the three farms. Specifically, PC1 clearly differentiated CA EOs from PE and PR EOs, which were positively projected and slightly separated from each other. On the other hand, PC2 attempted to maximize the differences within the other two farm groups by positively and negatively projecting PE and PR, respectively. Therefore, the composition of CA EOs seemed to be extremely different from those of PE and PR, confirming the results of the ANOVA analysis on terpenes abundance in EOs (Table S1).

PCA loading plots (Figure 1C,D) revealed which variables of EO composition (terpenes) induced the separation and differentiation of the samples basing on the farm group. In particular, the terpenes located on positive values of PC1 (Figure 1C) were higher in the EOs of PR > PE > CA, according to the results in Table 1. On the contrary, the terpenes located on negative values of PC1 displayed higher abundance percentages in CA EOs. Finally, terpenes negatively and positively projected on PC2 showed higher percentages for PR and PE groups, respectively.

Regarding the type of biostimulant treatment applied on lavandin plants, PCA did not reveal any clear separation of the samples in terms of EO compositions (Figure 1B). Indeed, the samples belonging to the same treatment group were not clustered and divided from the others within the same farm, and the comparable chemical compositions of the EOs might be due to the high inter-plant variability. In the case of PR EOs, even though marginally, CTRL EOs appeared separated from the other groups, while CTRL water and T2 treatments were similar, accordingly with p values in Table S2. So far, few studies have been made on the application of biostimulants on aromatic plants. To the best of our knowledge, none aimed to evaluate the changes in the chemical composition of lavandin EOs upon biostimulants application. Silva et al. [38] showed a small variation in the chemical composition of *Lavandula dentata* L. between the field and greenhouse experiments, and lavender plants fertilized with organo-mineral fertilizers perform better than or similar to plants fertilized with mineral fertilizers. Indeed, organo-mineral fertilizer can ensure a greater increase in yield due to the slow release of nutrients around the root system, especially at the flowering stage [38]. Foroutan Nia et al. [13] demonstrated that the fertilization of *Salvia rosmarinus* L. with AAs increases the ratios of the major compounds of the EO. Moreover, the authors showed that the obtained effect is dose-dependent and might vary according to the amount of nitrogen, potassium, phosphorous, and organic components present in the biostimulants [13]. Similar results were also obtained on parsley EO (*Petroselinum crispum* Mill.) by Mofeed et al. [39]. Furthermore, the application of seaweed extracts on *Mentha × piperita* L. and *Ocimum basilicum* L. have been proved to increase the content of the major compounds, leading to the obtainment of EOs with higher antibacterial activities [40]. In the present study, no significant changes have been observed in the ratios of the major compounds at the dosage and the developmental stage indicated by the supplier. However, the family, the genus, and the life cycle type of the aromatic plant have to be considered. Indeed, it is well-known that rosemary and basil are particularly subjected to chemical variations of the EO depending on the growing conditions.

The marked variation in the chemical composition of the EOs from the three farms might be due to several factors, and their impact on most of the biosynthetic pathways is still unclear. Several authors assert that cultivation techniques, in addition to pedo-climatic conditions, physicochemical characteristics of soil, and light exposure of the crop are crucial factors in the secondary metabolism of aromatic plants [41,42,43,44]. In our study, all lavandins belong to the same variety and were harvested under the same conditions and, therefore, the variation of composition might be due to both different cropping techniques and sunlight exposure of the aromatic plants [44]. Regarding the cropping conditions, population density, and space arrangement, have been demonstrated to represent a source of variation in plants’ growth, leading to noteworthy variations in plant biochemistry [45,46]. As an example, variations in both yield and chemical composition of peppermint Eos were reported in different plant populations [47]. Another important abiotic factor in the regulation of EO production is the sunlight exposition of the plants. Firstly, light acts on the activation of biosynthetic pathways of Eos by photosynthetic processes, and secondly, it increases environmental stress [48]. The following disruption of cellular homeostasis leads to the generation of reactive oxygen species (ROS) [49]. Furthermore, the prolonged exposure to full light results in an increase in temperature and soil drought. As a consequence, plants activate terpene biosynthetic pathways to face adverse conditions [50]. As an example, intense sunlight has been demonstrated to be directly related to the increased production of volatiles in *Anethum graveolens*, *Artemisia dracunculus*, and *Ocimum basilicum* [51]. These findings might explain the higher EO yield in PR and PE lavandins, which are south- and east-facing, respectively. On the contrary, CA lavandins, exposed to north-west, exhibited the lowest EO content. In addition, light exposure modulates the terpenes production and the corresponding transcripts to hinder ROS and protect plant physiology [52]. The variation of EO composition depends upon the type of plant species and unfortunately information regarding the effects of light exposure on *Lavandula* genus are missing. Thus, the observed differences in EO composition within lavandins from the three farms cannot be explained with certainty. It might be hypothesized that the variance of monoterpene abundances in the three main group of EOs is due to both environmental factors and cropping techniques, being all the other factors constant (Table S3).

In order to better understand the differences among the farms in the biosynthetic pathways, the percent compositions of the main terpene classes were calculated (Table 2). In particular, CA and PE EOs displayed high content of alcoholic monoterpenes (around 69% and 42%, respectively) and lower content of esters (around 6% and 33%, respectively). On the contrary, in the case of PR EOs, the ratio between these two classes appeared balanced, with the same percentage distribution (about 36%). In addition, ether, ketone/aldehyde, and sesquiterpene percentages resulted higher for PR and lower for CA, which exhibited the highest content of monoterpene hydrocarbons. In general, it seemed that in CA lavandin alcohol acetyltransferase (in particular, linalool and lavandulyl acetyltransferase) and oxidative enzymes (such as borneol dehydrogenase) were scarcely active as if it is at an early vegetative stage

### 2.3. Hydrosol Characterization

The terpenoids in hydrosol represented 0.067 ± 0.015% *w/w* of the total weight. The qualitative and semi-quantitative results from GC analysis of hydrosols are displayed in Table 3. The hydrosols showed high ratios of alcohols, mainly represented by linalool, borneol, α-terpineol, and terpinene-4-ol. Alcohols were the most concentrated terpenoids due to their high solubility in water. Moreover, part of them might derive from the molecular rearrangement of other terpenes during the extraction process. Indeed, the oxides of linalool and α-terpineol were more abundant in the hydrosols than in the EOs, as observed by Šilha et al. in *Lavandula angustifolia* and Baydar et al. in *Lavandula x intermedia* [31,53]. This evidence can be explained by the fact that in boiling water (during the steam distillation) linalool might undergo oxidations and cyclization processes [53]. Monoterpene hydrocarbons represented 1–2% of the whole volatile compounds, according to their limited solubility in the aqueous environment. Linalyl acetate, the other characterizing monoterpene of lavandin EO, was not detected in the hydrosol, probably due to its limited solubility and hydrolysis in water.

As in the case of EOs, hydrosols exhibited significant differences within the farms. Indeed, CA hydrosols stood out for all the ratios of the terpenes (*p* < 0.01), with the exception of linalool, lavandulol, and limonene. Regarding the other two farms, PR showed higher content of camphor (*p* < 0.01). As observed in the EOs, PE, and PR hydrosols displayed higher amounts of ketones than CA hydrosols.

The treatments did not induce significant differences in the relative percentages of the major components. On minor components, some dissimilarities were observed with p values lower than 0.05. In CA hydrosols, neomenthol and terpinene-4-ol were significantly lower in T1 and T2, respectively, than CTRL. PE groups resulted different for trans linalool oxide in T1, neomenthol in T2, and menthol in T1 and T2 compared to CTRL. Finally, PR hydrosols showed differences for trans linalool oxide in T1, camphor in T1 and T2 compared to CTRLw, and lavandulyl acetate in T2 versus CTRL.

## 3. Materials and Methods

### 3.1. Materials

The two biostimulants tested were: FITOSTIM^®^ and FITOSTIM^®^ ALGA (both marketed by the Italian company SCAM S.p.A., Modena, Italy). FITOSTIM^®^ is a biostimulant consisting of AA, peptides, and peptones. Characteristics shown on the label were: total organic nitrogen (N): 8.0%, organic carbon (C) of biological origin: 25.2%, C/N ratio: 3.15, the average molecular weight of hydrolysates: <2500 Dalton glycine/proline + hydroxyproline ratio: 1.1, dry hydrolysis degree: 380, and free amino acids: 15%. FITOSTIM^®^ ALGA contains brown marine algae (*Ascophyllum nodosum*) rich in betaines, vitamins, natural promoters, polysaccharides, and trace elements, and also, AA, peptides, and peptones. Characteristics shown on the label are: organic nitrogen (N): 2.0%, organic carbon (C) of biological origin: 10.0%, pH: 7.5–8.5, and organic substance with nominal molecular weight <50 kDa: 50%. The biostimulants were diluted with water at the final concentration of 0.15% *w/w* prior the application on crops.

Ethyl acetate (EtOAc), *n*-hexane (Hex), sodium sulphate anhydrous (Na_2_SO_4_), and C_8_-C_40_ n-Alkanes Calibration Standard were of analytical grade from Merck Life Science (Milan, Italy).

### 3.2. Growing Conditions and Experimental Design

The *Lavandula x intermedia* plants cv. “Grosso” were cultivated in three different farms in Emilia-Romagna Region Apennines (Table S3): Campazzo (CA), Montombraro of Zocca, Modena, Italy (9X4J+7W map), north-west-facing; Pedroni Paola (PE), Zocca, Modena, Italy, (9X2V+2C map) with east-facing; Preci Carlo (PR), Villa d’Aiano, Castel d’Aiano, Bologna, Italy (7XWH+3F map), south-facing. The crops in all the farms did not receive any fertilizer, irrigation, or plant protection product.

Lavandin crops were transplanted in 2013, 2009, and 2016 in CA, PE, and PR, respectively. In CA and PE locations, the plant density was the same, with a spacing of 170 cm between rows and 50 cm between plants in the row. In PR farm, the plant density was lower, with a spacing of 140 cm between rows and 80 cm between plants in the same row.

Lavandin plants were assigned into one of four treatments: farm control (CTRL), where no treatment was applied; foliar application of water (CTRL w); foliar application of FITOSTIM (T1); foliar application of FITOSTIM ALGA (T2). The experimental design was completely randomized, with three replications and each replication contained 2 plants. The foliar application of biostimulants (0.5 L/plant) was performed at beginning of blooming on 13 June 2020, and repeated after 14 days before the full blooming. The liquid solutions were distributed by nebulization using a hand pressure sprayer.

The aerial parts of lavandin plants were hand-picked (harvesting all inflorescences) on 11 July 2020 morning, when inflorescences were in full blooming. The fresh biomass of each plant was determined in order to calculate the quantity of EOs per plant as follows: EOs/plant = Fresh biomass/plant (g) * EOs yield (%).

### 3.3. Steam Distillation

Lavender EOs were extracted from fresh aerial parts through steam distillation, according to the European Pharmacopoeia X Ed. [54]. About 250–300 g of flowers were steam distilled for 1 h by a stainless-steel distiller (Albrigi Luigi s.r.l., Stallavena, VR, Italy). The EOs were collected in a Clevenger-type apparatus (Albrigi Luigi s.r.l.). The EO in upper layer was separated by hydrosol and measured on an analytical scale. The percent yield of the EOs was calculated as weight of oil per weight of fresh lavender flowers. The EOs were stored at 4 °C until analysis.

### 3.4. Hydrosol Extraction

Hydrosol has been subjected to liquid-liquid extraction in order to characterize its chemical composition. Briefly, 10 g of hydrosol were extracted at first with EtOAc (3 × 5 mL); subsequently with Hex) (3 × 5 mL), and the combined organic phases were washed with brine. The organic phase was dried over Na_2_SO_4_ and concentrated at room temperature under vacuum. The residue was weighted, solubilized in Hex, and analyzed by GC.

### 3.5. Analysis

#### 3.5.1. GC-MS Analysis

Analyses were performed on a 7890A gas chromatograph coupled with a 5975C network mass spectrometer (GC-MS) (Agilent Technologies, Milan, Italy). Compounds were separated on an Agilent Technologies HP-5 MS cross-linked poly-5% diphenyl–95% dimethyl polysiloxane (30 m × 0.25 mm i.d., 0.25 μm film thickness) capillary column, according to a gradient temperature program to obtain better separation of the peaks and to allow the complete elution of all components. The column temperature was initially set at 45 °C, then increased at a rate of 2 °C/min up to 100 °C, then raised to 250 °C at a rate of 5 °C/min, and, finally, held for 5 min. The injection volume was 0.1 μL, with a split ratio 1:20. Helium was used as the carrier gas, at a flow rate of 0.7 mL/min. The injector, transfer line, and ion-source temperature was 250, 280, and 230 °C, respectively. MS detection was performed with electron ionization (EI) at 70 eV, operating in the full-scan acquisition mode in the m/z range 40–400. The EOs were diluted 1:20 (*v/v*) with *n*-hexane before GC-MS analysis.

#### 3.5.2. GC-FID Analysis

Chromatographic characterization of EOs was performed on a 7820 gas chromatograph (Agilent Technologies, Milan, Italy) with a flame ionization detector (FID). EOs and the mixture of aliphatic hydrocarbons (C_8_–C_40_) were diluted 1:20 (*v/v*) with Hex before GC-FID analysis. Helium was used as carrier gas at a flow rate of 1 mL/min with a pressure of 2.5 bar at the column head. The injector and detector temperatures were set at 250 and 300 °C, respectively. EO components were separated on an Agilent Technologies HP-5 crosslinked poly-5% diphenyl–95% dimethyl siloxane (30 m × 0.32 mm i.d., 0.25 mm film thickness) capillary column. The column temperature was initially set at 45 °C, then increased at a rate of 2 °C/min up to 100 °C, then raised to 250 °C at a rate of 5 °C/min, and, finally, maintained for 5 min. The injection volume was 1 μL, with a split ratio 1:20.

Compounds were identified by comparing the retention times of the chromatographic peaks with those of authentic reference standards run under the same conditions and by comparing the linear retention indices (*LRI*s) relative to C_8_-C_40_ *n*-alkanes obtained on the HP-5 column under the above-mentioned conditions with the literature [55]. Peak enrichment by co-injection with authentic reference compounds was also carried out. Comparison of the MS-fragmentation pattern of the target analytes with those of pure components was performed, by using the National Institute of Standards and Technology (NIST version 2.0d, 2005) mass-spectral database.

The percentage relative amount of individual components was expressed as the percent peak area relative to the total peak area obtained by the GC/FID analysis. Semi-quantitative data were acquired from the mean of two analyses. The percentages of each compound are expressed as the mean ± standard deviation (SD) of the three replicates for each kind of treatment.

The data acquisition and processing were performed using the OpenLab CDS C.01.04 (Agilent Technologies, Santa Clara, CA, USA) software.

### 3.6. Statistical Analyses

The PCA was performed by using PLS_Toolbox 8.9.2 software (Eigenvector Research Inc., Manson, WA, USA) for MATLAB^®^ on mean-centered data from semi-quantitative analysis in GC-FID. Two-way ANOVA followed by Tukey’s post-hoc test was performed on GraphPad Prism 8.4.3 (GraphPad Software, Inc., San Diego, CA, USA) on the yield percentages from steam distillation.

One-way multivariate analysis of variance (MANOVA) followed by Tukey’s post-hoc test was performed on SPSS (version 8.3, San Diego, CA, USA). MANOVA was used to identify significant differences (*p* > 0.05) within crop treatments and farms on the relative abundance EO components. The normal distribution of data was checked with Shapiro–Wilk test to evaluate the correct descriptive statistics and statistical tests to be employed.

## 4. Conclusions

To the best of our knowledge, this is the first research work which aimed to evaluate the effects of biostimulants on the composition of *Lavandula x intermedia* (Emeric ex Loisel.) EOs and hydrosols. The compositional results of the EOs showed that biostimulants did not induce any change in terpene ratios and EO yields; however, the EO per plant parameter greatly benefited of the biostimulant treatment. Findings of the present work suggest that both animal- or plant-based biostimulants might be successfully employed to sustain lavandin crops and to increase the biomass production without endangering the characteristic chemical composition of the produced EO. On the contrary, huge differences were noticed within the three farms included in the trial. These differences seemed to be related to sunlight exposure and different maintenance of the crops over the years. However, due to the scarcity of studies on the abiotic factors affecting the composition of *Lavandula* genus EOs in the literature, firm conclusions could not be drawn. Further studies are ongoing to assess the effect of biostimulants on the agronomic parameters in different years and meteorological conditions.

## Figures and Tables

**Table 1:** The yields of the steam distilled essential oils (mean ± standard deviation of each sample group, n = 3) expressed as percentages (*w/w*) and g per plant. Distinct letters were used to differentiate statistically different groups according to Tukey’s post-hoc test (*p* < 0.05).

Farm	Treatment	Yield *w/w* % (Mean ± SD)		Yield g per Plant (Mean ± SD)	
CA	CTRL	1.04 ± 0.04	a	4.14 ± 0.49	d
CA	CTRL W	1.30 ± 0.20	a	6.38 ± 0.52	d
CA	T1	1.11 ± 0.13	a	5.22 ± 1.22	d
CA	T2	1.22 ± 0.29	a	5.28 ± 0.99	d
PE	CTRL	2.42 ± 0.24	b	3.97 ± 0.47	d
PE	CTRL W	2.32 ± 0.20	b	3.90 ± 1.21	d
PE	T1	2.48 ± 0.08	b	5.09 ± 0.64	d
PE	T2	2.60 ± 0.10	b	4.40 ± 0.58	d
PR	CTRL	2.66 ± 0.03	b	13.23 ± 1.46	c
PR	CTRL W	2.80 ± 0.13	b	15.04 ± 2.81	bc
PR	T1	2.45 ± 0.43	b	18.44 ± 3.63	ab
PR	T2	2.34 ± 0.06	b	21.03 ± 0.95	a

CA, Campazzo farm; PE, Pedroni farm; PR, Preci farm; CTRL, farm control; CTRL W, foliar application of water; T1, foliar application of FITOSTIM; T2, foliar application of FITOSTIM ALGA.

**Table 2:** Chemical composition % of EOs expressed as mean ± standard deviation.

		CA				PE				PR			
	*LRI*	CTRL	CTRL W	T1	T2	CTRL	CTRL W	T1	T2	CTRL	CTRL W	T1	T2
2-hexenal	865	0.34 ± 0.13	0.36 ± 0.05	0.33 ± 0.09	0.35 ± 0.11	-	-	-	-	-	-	-	-
α-thujene	925	0.13 ± 0.03	0.13 ± 0.01	0.12 ± 0.01	0.15 ± 0.03	-	-	-	-	-	-	-	-
α-pinene	932	0.54 ± 0.07	0.54 ± 0.02	0.51 ± 0.04	0.58 ± 0.11	0.48 ± 0.09	0.46 ± 0.05	0.43 ± 0.04	0.41 ± 0.03	0.49 ± 0.05	0.61 ± 0.08	0.58 ± 0.05	0.61 ± 0.09
Camphene	946	0.46 ± 0.02	0.47 ± 0.02	0.45 ± 0.02	0.50 ± 0.10	0.35 ± 0.05	0.34 ± 0.02	0.32 ± 0.03	0.30 ± 0.02	0.34 ± 0.02	0.36 ± 0.03	0.36 ± 0.01	0.38 ± 0.05
Sabinene	972	0.16 ± 0.04	0.16 ± 0.02	0.15 ± 0.01	0.19 ± 0.05	0.14 ± 0.02	0.13 ± 0.02	0.13 ± 0.01	0.12 ± 0.01	0.15 ± 0.02	0.21 ± 0.02	0.19 ± 0.02	0.21 ± 0.01
β-pinene	975	0.15 ± 0.03	0.15 ± 0.01	0.15 ± 0.01	0.17 ± 0.02	0.45 ± 0.09	0.45 ± 0.07	0.43 ± 0.03	0.40 ± 0.04	0.49 ± 0.09	0.69 ± 0.09	0.61 ± 0.07	0.65 ± 0.08
oct-1-en-3-ol	978	0.91 ± 0.09	0.97 ± 0.06	0.82 ± 0.05	0.99 ± 0.07	0.29 ± 0.04	0.27 ± 0.02	0.23 ± 0.06	0.23 ± 0.02	0.25 ± 0.05	0.18 ± 0.08	0.14 ± 0.00	0.19 ± 0.09
Myrcene	991	1.14 ± 0.17	1.08 ± 0.08	1.02 ± 0.13	1.20 ± 0.21	1.65 ± 0.74	1.43 ± 0.25	1.16 ± 0.23	1.08 ± 0.05	1.28 ± 0.15	1.17 ± 0.23	1.30 ± 0.23	1.32 ± 0.23
α-phellandrene	1004	0.15 ± 0.02	0.14 ± 0.01	0.14 ± 0.02	0.15 ± 0.03	0.07 ± 0.01	0.08 ± 0.01	0.07 ± 0.01	0.06 ± 0.00	0.07 ± 0.00	0.08 ± 0.00	0.08 ± 0.00	0.08 ± 0.01
δ-3-carene	1009	0.26 ± 0.07	0.25 ± 0.03	0.25 ± 0.01	0.30 ± 0.09	0.11 ± 0.02	0.11 ± 0.02	0.10 ± 0.01	0.09 ± 0.01	0.14 ± 0.03	0.19 ± 0.04	0.17 ± 0.02	0.17 ± 0.04
α-terpinene	1015	0.21 ± 0.05	0.20 ± 0.02	0.20 ± 0.04	0.23 ± 0.05	0.20 ± 0.07	0.24 ± 0.01	0.19 ± 0.02	0.17 ± 0.03	0.16 ± 0.02	0.20 ± 0.04	0.17 ± 0.02	0.19 ± 0.04
*p*-cymene	1023	0.08 ± 0.02	0.08 ± 0.01	0.08 ± 0.01	0.10 ± 0.03	-	-	-	-	-	-	-	-
Limonene	1028	5.68 ± 1.43	5.38 ± 0.59	5.09 ± 0.38	6.46 ± 2.06	0.96 ± 0.16	1.02 ± 0.12	0.84 ± 0.11	0.80 ± 0.03	0.93 ± 0.10	0.86 ± 0.13	0.90 ± 0.07	0.74 ± 0.59
1,8-cineole	1031	2.83 ± 0.29	2.85 ± 0.10	3.04 ± 0.22	3.25 ± 0.15	4.61 ± 0.49	4.57 ± 0.62	4.69 ± 0.19	4.27 ± 0.34	4.75 ± 0.71	6.42 ± 0.44	5.76 ± 0.30	6.48 ± 0.57
cis-ocimene	1038	4.36 ± 0.65	4.16 ± 0.31	4.02 ± 0.31	4.75 ± 1.17	1.43 ± 0.27	1.39 ± 0.08	1.19 ± 0.11	1.14 ± 0.04	1.54 ± 0.12	1.37 ± 0.08	1.58 ± 0.15	1.62 ± 0.16
trans-ocimene	1048	1.47 ± 0.31	1.28 ± 0.14	1.39 ± 0.17	1.39 ± 0.29	0.73 ± 0.22	0.76 ± 0.14	0.58 ± 0.13	0.53 ± 0.03	0.65 ± 0.09	0.55 ± 0.13	0.63 ± 0.13	0.66 ± 0.12
γ-terpinene	1058	0.26 ± 0.05	0.25 ± 0.03	0.25 ± 0.05	0.26 ± 0.06	0.13 ± 0.03	0.14 ± 0.02	0.12 ± 0.02	0.11 ± 0.01	0.13 ± 0.02	0.13 ± 0.02	0.14 ± 0.01	0.13 ± 0.03
trans-sabinene hydrate	1065	0.13 ± 0.05	0.13 ± 0.07	0.12 ± 0.05	0.17 ± 0.05	0.09 ± 0.03	0.08 ± 0.02	0.09 ± 0.02	0.11 ± 0.03	0.11 ± 0.02	0.15 ± 0.05	0.13 ± 0.02	0.16 ± 0.05
cis-linalool oxide	1072	0.14 ± 0.00	0.13 ± 0.01	0.13 ± 0.01	0.13 ± 0.01	0.15 ± 0.01	0.15 ± 0.01	0.15 ± 0.01	0.15 ± 0.02	0.14 ± 0.01	0.13 ± 0.00	0.15 ± 0.02	0.15 ± 0.02
trans-linalool oxide	1087	0.56 ± 0.06	0.50 ± 0.02	0.51 ± 0.05	0.54 ± 0.09	0.48 ± 0.06	0.49 ± 0.04	0.44 ± 0.04	0.42 ± 0.02	0.45 ± 0.02	0.42 ± 0.03	0.44 ± 0.01	0.45 ± 0.05
Linalool	1108	46.98 ± 3.01	50.21 ± 2.16	49.45 ± 0.82	46.61 ± 6.86	33.43 ± 1.00	33.87 ± 1.53	33.72 ± 0.83	32.58 ± 0.59	31.05 ± 0.25	27.82 ± 1.24	29.73 ± 1.33	28.99 ± 1.19
Fenchol	1115	-	-	-	-	0.55 ± 0.05	0.52 ± 0.03	0.42 ± 0.12	0.45 ± 0.02	0.40 ± 0.16	0.35 ± 0.18	0.23 ± 0.01	0.32 ± 0.18
trans-rose oxide	1130	0.14 ± 0.01	0.17 ± 0.07	0.13 ± 0.02	0.15 ± 0.04	-	-	-	-	-	-	-	-
Camphor	1144	2.22 ± 0.17	1.74 ± 0.18	1.94 ± 0.14	2.17 ± 0.68	6.62 ± 0.13	6.43 ± 0.55	6.30 ± 0.18	6.31 ± 0.44	6.97 ± 0.14	7.67 ± 0.16	7.40 ± 0.18	7.70 ± 0.21
trans-verbenol	1151	0.15 ± 0.02	0.17 ± 0.01	0.15 ± 0.00	0.17 ± 0.03	0.11 ± 0.01	0.10 ± 0.01	0.09 ± 0.00	0.10 ± 0.01	0.09 ± 0.00	0.09 ± 0.01	0.10 ± 0.01	0.11 ± 0.01
Borneol	1167	11.65 ± 0.77	11.09 ± 1.31	11.86 ± 0.86	10.95 ± 1.81	3.78 ± 0.30	3.98 ± 0.50	4.15 ± 0.17	3.98 ± 0.31	3.01 ± 0.02	2.60 ± 0.16	2.55 ± 0.03	2.46 ± 0.15
lavandulol	1171	0.66 ± 0.09	0.55 ± 0.08	0.63 ± 0.09	0.70 ± 0.04	0.45 ± 0.02	0.51 ± 0.05	0.45 ± 0.04	0.46 ± 0.01	0.48 ± 0.03	0.38 ± 0.01	0.38 ± 0.00	0.37 ± 0.03
Terpinen-4-ol	1181	5.58 ± 0.64	5.30 ± 0.37	5.29 ± 0.35	5.35 ± 0.79	1.95 ± 0.02	1.95 ± 0.06	1.96 ± 0.05	1.98 ± 0.16	2.05 ± 0.05	1.90 ± 0.09	2.07 ± 0.04	1.97 ± 0.05
*p*-cymen-8-ol	1187	0.30 ± 0.03	0.27 ± 0.03	0.29 ± 0.03	0.35 ± 0.15	-	-	-	-	-	-	-	-
α-terpineol	1191	0.29 ± 0.06	0.25 ± 0.03	0.26 ± 0.04	0.27 ± 0.01	0.71 ± 0.17	0.72 ± 0.12	0.71 ± 0.14	0.64 ± 0.08	0.70 ± 0.10	0.78 ± 0.13	0.67 ± 0.08	0.78 ± 0.13
Myrtenal	1194	0.49 ± 0.04	0.52 ± 0.02	0.52 ± 0.02	0.50 ± 0.04	0.25 ± 0.01	0.25 ± 0.02	0.24 ± 0.01	0.24 ± 0.01	0.20 ± 0.01	0.18 ± 0.01	0.21 ± 0.02	0.20 ± 0.01
Nerol	1230	0.16 ± 0.01	0.16 ± 0.02	0.15 ± 0.01	0.16 ± 0.01	0.09 ± 0.03	0.09 ± 0.02	0.09 ± 0.03	0.07 ± 0.01	0.08 ± 0.02	0.09 ± 0.02	0.07 ± 0.01	0.08 ± 0.03
Pulegone	1242	0.34 ± 0.03	0.32 ± 0.01	0.31 ± 0.01	0.37 ± 0.09	-	-	-	-	-	-	-	-
Carvone	1247	0.14 ± 0.00	0.14 ± 0.00	0.14 ± 0.00	0.15 ± 0.01	0.13 ± 0.00	0.12 ± 0.00	0.12 ± 0.01	0.13 ± 0.01	0.11 ± 0.00	0.11 ± 0.00	0.11 ± 0.01	0.11 ± 0.01
Linalyl acetate	1263	5.25 ± 0.82	4.70 ± 0.42	4.75 ± 0.29	4.81 ± 0.84	30.16 ± 2.26	29.89 ± 1.75	30.94 ± 0.80	32.60 ± 0.49	32.02 ± 1.62	33.04 ± 2.00	33.17 ± 1.27	32.42 ± 1.50
Lavandulyl acetate	1293	1.27 ± 0.10	1.10 ± 0.11	1.23 ± 0.01	1.19 ± 0.24	2.87 ± 0.17	2.94 ± 0.10	2.88 ± 0.02	2.97 ± 0.12	3.12 ± 0.17	3.06 ± 0.12	2.64 ± 0.04	2.75 ± 0.18
Neryl acetate	1367	0.07 ± 0.01	0.05 ± 0.01	0.05 ± 0.01	0.05 ± 0.00	0.29 ± 0.06	0.30 ± 0.05	0.26 ± 0.05	0.24 ± 0.02	0.28 ± 0.05	0.25 ± 0.05	0.26 ± 0.05	0.28 ± 0.06
α-copaene	1383	-	-	-	-	0.09 ± 0.00	0.09 ± 0.01	0.09 ± 0.00	0.09 ± 0.00	0.11 ± 0.01	0.13 ± 0.01	0.11 ± 0.01	0.12 ± 0.01
β-cubebene	1386	0.10 ± 0.04	0.07 ± 0.02	0.07 ± 0.02	0.08 ± 0.01	0.60 ± 0.13	0.65 ± 0.10	0.54 ± 0.09	0.54 ± 0.03	0.61 ± 0.09	0.56 ± 0.12	0.57 ± 0.11	0.58 ± 0.10
Geranyl acetate	1424	0.14 ± 0.01	0.13 ± 0.02	0.14 ± 0.00	0.15 ± 0.04	-	-	-	-	-	-	-	-
β-caryophyllene	1439	0.47 ± 0.06	0.41 ± 0.05	0.44 ± 0.01	0.46 ± 0.08	1.55 ± 0.08	1.62 ± 0.15	1.47 ± 0.12	1.57 ± 0.06	1.78 ± 0.09	1.87 ± 0.13	1.72 ± 0.19	1.81 ± 0.18
α-bergamotene	1460	0.06 ± 0.00	0.05 ± 0.00	0.05 ± 0.00	0.07 ± 0.01	0.12 ± 0.01	0.13 ± 0.01	0.12 ± 0.01	0.13 ± 0.01	0.16 ± 0.02	0.18 ± 0.02	0.16 ± 0.02	0.16 ± 0.02
β-farnesene	1487	2.15 ± 0.14	1.99 ± 0.32	2.18 ± 0.04	2.23 ± 0.48	1.13 ± 0.13	1.18 ± 0.15	1.17 ± 0.07	1.26 ± 0.06	1.30 ± 0.07	1.33 ± 0.07	1.24 ± 0.11	1.24 ± 0.17
ar-curcumene	1511	0.20 ± 0.02	0.16 ± 0.04	0.18 ± 0.02	0.20 ± 0.07	0.66 ± 0.10	0.65 ± 0.07	0.68 ± 0.05	0.75 ± 0.04	0.88 ± 0.08	0.92 ± 0.01	0.87 ± 0.10	0.86 ± 0.14
δ-cadinene	1521	0.49 ± 0.12	0.46 ± 0.10	0.46 ± 0.04	0.48 ± 0.12	0.52 ± 0.08	0.55 ± 0.11	0.53 ± 0.04	0.58 ± 0.05	0.58 ± 0.05	0.53 ± 0.15	0.58 ± 0.06	0.60 ± 0.10
γ-cadinene	1529	-	-	-	-	0.36 ± 0.05	0.39 ± 0.07	0.34 ± 0.03	0.38 ± 0.03	0.32 ± 0.07	0.40 ± 0.12	0.29 ± 0.06	0.34 ± 0.07
Total		99.36 ± 0.36	99.3 ± 0.36	99.52 ± 0.18	99.51 ± 0.37	98.47 ± 0.42	98.71 ± 0.42	98.16 ± 0.29	98.11 ± 0.23	98.07 ± 0.31	97.64 ± 0.24	98.23 ± 0.44	98.18 ± 0.24
monoterpene hydrocarbons		15.4 ± 3.03	14.63 ± 1.22	14.16 ± 1.25	16.77 ± 4.00	6.7 ± 1.73	6.54 ± 0.69	5.58 ± 0.69	5.22 ± 0.28	6.35 ± 0.5	6.42 ± 0.83	6.71 ± 0.75	6.77 ± 1.12
ethers		3.66 ± 0.35	3.66 ± 0.18	3.81 ± 0.17	4.07 ± 0.24	5.24 ± 0.55	5.22 ± 0.63	5.28 ± 0.22	4.83 ± 0.37	5.33 ± 0.72	6.97 ± 0.46	6.35 ± 0.28	7.09 ± 0.58
alcohols		66.82 ± 3.03	69.09 ± 1.37	69.03 ± 0.65	65.72 ± 5.97	41.46 ± 0.92	42.09 ± 1.14	41.9 ± 0.96	40.58 ± 0.59	38.22 ± 0.31	34.34 ± 1.59	36.07 ± 1.26	35.43 ± 1.29
ketones and aldehydes		3.18 ± 0.17	2.72 ± 0.15	2.9 ± 0.11	3.19 ± 0.74	7 ± 0.12	6.81 ± 0.56	6.67 ± 0.18	6.67 ± 0.45	7.29 ± 0.15	7.97 ± 0.15	7.72 ± 0.18	8.01 ± 0.2
esters		6.68 ± 0.89	5.93 ± 0.52	6.11 ± 0.28	6.13 ± 1.09	33.39 ± 2.38	33.19 ± 1.71	34.14 ± 0.76	35.88 ± 0.58	35.47 ± 1.75	36.41 ± 2.04	36.14 ± 1.24	35.52 ± 1.65
sesquiterpenes		3.61 ± 0.35	3.27 ± 0.51	3.52 ± 0.04	3.65 ± 0.75	4.67 ± 0.31	4.86 ± 0.57	4.59 ± 0.32	4.93 ± 0.16	5.41 ± 0.37	5.53 ± 0.23	5.25 ± 0.58	5.37 ± 0.66

CA, Campazzo farm; PE, Pedroni farm; PR, Preci farm; CTRL, farm control; CTRL W, foliar application of water; T1, foliar application of FITOSTIM; T2, foliar application of FITOSTIM ALGA.

**Table 3:** Hydrosol terpene % composition of all the samples expressed as mean ± standard deviation.

	LRI	CA				PE				PR			
		CTRL az	CTRL H2O	T1	T2	CTRL az	CTRL H2O	T1	T2	CTRL az	CTRL H2O	T1	T2
β-pinene	978	0.56 ± 0.04	0.98 ± 0.07	0.81 ± 0.09	0.84 ± 0.39	0.39 ± 0.06	0.42 ± 0.05	0.27 ± 0.06	0.22 ± 0.01	0.47 ± 0.15	0.22 ± 0.08	0.19 ± 0.01	0.35 ± 0.01
1,8-cineole	1029	0.46 ± 0.10	0.6 ± 0.14	0.86 ± 0.23	1.17 ± 0.04	1.16 ± 1.47	0.7 ± 0.12	0.8 ± 0.3	0.77 ± 0.1	0.44 ± 0.2	1.10 ± 0.21	0.6 ± 0.40	0.4 ± 0.02
cis-linalool oxide	1071	0.7 ± 0.04	0.57 ± 0.06	0.65 ± 0.01	0.64 ± 0.17	2.08 ± 0.58	1.74 ± 0.17	2.31 ± 0.18	2.17 ± 0.14	1.55 ± 0.18	2.24 ± 0.67	2.36 ± 0.88	2.11 ± 0.19
trans-linalool oxide	1087	0.82 ± 0.05	0.57 ± 0.06	0.62 ± 0.02	0.67 ± 0.11	2.14 ± 0.53	1.74 ± 0.15	2.47 ± 0.23	2.32 ± 0.2	1.59 ± 0.2	2.31 ± 0.67	2.49 ± 0.85	2.13 ± 0.3
Linalool	1101	34.23 ± 1.72	43.81 ± 4.72	39.6 ± 3.95	42.12 ± 10.33	43.2 ± 10.3	47.5 ± 0.83	41.53 ± 2.73	40.3 ± 2.68	46.28 ± 2.56	38.74 ± 2.84	43.1 ± 1.44	39.12 ± 0.14
Camphor	1143	3.27 ± 0.29	2.76 ± 0.01	3.55 ± 0.51	3.06 ± 0.15	11.78 ± 0.53	14.78 ± 1.18	12.8 ± 0.68	11.8 ± 2.67	15.11 ± 0.53	18.76 ± 0.88	15.44 ± 0.02	13.36 ± 1.68
Borneol	1166	31.94 ± 1.65	26.85 ± 2.14	30.82 ± 3.21	30.49 ± 7.93	12.3 ± 1.13	13.09 ± 0.64	14.25 ± 0.29	14.43 ± 1.41	11.59 ± 0.15	10.64 ± 0.69	10.04 ± 0.63	10.31 ± 0.17
Neomenthol	1168	2.51 ± 0.62	1.93 ± 0.92	2.04 ± 0.44	1.97 ± 0.35	1.77 ± 0.10	1.79 ± 0.16	2.05 ± 0.2	2.2 ± 0.27	1.8 ± 0.13	1.73 ± 0.11	1.79 ± 0.06	1.86 ± 0.07
Lavandulol	1175	0.25 ± 0.56	0.19 ± 0.21	0.19 ± 0.1	0.19 ± 0.09	0.52 ± 0.09	0.4 ± 0.07	0.63 ± 0.16	0.7 ± 0.16	0.42 ± 0.06	0.49 ± 0.10	0.59 ± 0.09	0.65 ± 0.06
Terpinen-4-ol	1178	10.97 ± 1.47	8.7 ± 0.54	9.6 ± 0.68	8.87 ± 0.55	5.12 ± 0.67	4.8 ± 0.04	4.88 ± 0.09	5.22 ± 0.34	5.43 ± 0.06	5.3 ± 0.13	6.02 ± 0.06	5.4 ± 0.14
Menthol	1183	0.55 ± 0.03	0.29 ± 0.04	0.42 ± 0.09	0.33 ± 0.12	0.55 ± 0.12	0.5 ± 0.06	0.78 ± 0.12	0.81 ± 0.09	0.5 ± 0.01	0.61 ± 0.06	0.61 ± 0.02	0.7 ± 0.11
*p*-cymen-8-ol	1186	3.69 ± 0.5	2.23 ± 0.2	2.85 ± 0.6	2.66 ± 1.26	0.7 ± 0.08	0.67 ± 0.11	0.97 ± 0.23	1.12 ± 0.26	0.81 ± 0.03	1.06 ± 0.07	0.84 ± 0.06	1.05 ± 0.13
α-terpineol	1190	2.53 ± 0.52	1.71 ± 0.38	1.92 ± 0.1	1.93 ± 0.23	7 ± 1.62	7.04 ± 0.19	7.66 ± 0.73	8.3 ± 0.18	7.39 ± 0.03	7.8 ± 0.10	7.88 ± 0.94	8.73 ± 1.17
Nerol	1232	0.73 ± 0.11	0.44 ± 0.1	0.59 ± 0.08	0.48 ± 0.05	1.09 ± 0.18	1.06 ± 0.1	1.13 ± 0.22	1.29 ± 0.05	1.12 ± 0.04	0.88 ± 0.06	1.08 ± 0.17	0.99 ± 0.09
Piperitone	1259	0.86 ± 0.43	0.65 ± 0.02	0.74 ± 0.06	0.55 ± 0.18	2.48 ± 1.06	1.83 ± 0.25	2.51 ± 0.47	2.87 ± 0.01	2.05 ± 0.36	2.12 ± 0.14	2.12 ± 0.36	2.55 ± 0.33
Lavandulyl acetate	1292	0.69 ± 0.23	0.27 ± 0.07	0.67 ± 0.3	0.51 ± 0.4	0.25 ± 0.10	0.25 ± 0.03	0.37 ± 0.07	0.36 ± 0.07	0.25 ± 0.1	0.34 ± 0.01	0.56 ± 0.33	0.96 ± 0.85
Total		91.56 ± 2.35	95.9 ± 2.01	93.53 ± 1.47	94.2 ± 1.19	83.53 ± 6.49	91.31 ± 1.11	87.3 ± 1.61	85.51 ± 2.38	89.16 ± 2.83	87.47 ± 1.66	87.84 ± 2.02	82.23 ± 2.38
monoterpene hydrocarbons		0.56 ± 0.04	0.98 ± 0.07	0.81 ± 0.09	0.84 ± 0.39	0.39 ± 0.06	0.42 ± 0.05	0.27 ± 0.06	0.22 ± 0.01	0.47 ± 0.15	0.22 ± 0.08	0.19 ± 0.01	0.35 ± 0.01
ethers		2 ± 0.09	1.73 ± 0.09	2.19 ± 0.19	2.4 ± 0.26	5.98 ± 2.1	4.08 ± 0.36	5.61 ± 0.09	4.83 ± 0.17	3.72 ± 0.47	5.71 ± 1.09	5.95 ± 1.74	4.63 ± 0.63
alcohols		84.4 ± 1.68	89.5 ± 1.98	85.94 ± 0.56	86.83 ± 0.96	62.42 ± 9.79	69.75 ± 0.22	65.75 ± 1.64	66.05 ± 0.90	67.41 ± 1.82	58.69 ± 2.63	63.58 ± 1.7	60 ± 0.24
ketones		3.96 ± 0.59	3.42 ± 0.59	4.39 ± 0.36	3.62 ± 0.03	14.64 ± 1.29	16.83 ± 0.75	15.27 ± 0.17	14.04 ± 2.17	17.37 ± 0.72	21.12 ± 0.84	17.48 ± 0.31	16.22 ± 1.1
esters		0.69 ± 0.23	0.27 ± 0.07	0.67 ± 0.3	0.51 ± 0.4	0.25 ± 0.10	0.25 ± 0.03	0.37 ± 0.07	0.36 ± 0.07	0.25 ± 0.1	0.34 ± 0.01	0.56 ± 0.33	0.96 ± 0.85

## Data Availability

The data presented in this study are available on request from the corresponding author.

## Supplementary Materials

The following are available online. Table S1: One-way ANOVA results of significant differences between farms. Table S2: One-way ANOVA results of significant differences between treatments in farms. Table S3: Weather conditions and soil parameter of the investigated farms.
