# Supplementary material for: Effects of Biostimulants on the Chemical Composition of Essential Oil and Hydrosol of Lavandin (Lavandula x intermedia Emeric ex Loisel.) Cultivated in Tuscan-Emilian Apennines
Source: Molecules. 2021 Oct 12;26(20):6157. doi: 10.3390/molecules26206157 (PMC8537348; doi:10.3390/molecules26206157)
Supplement: Supplementary file 1 [file molecules-26-06157-s001.zip › molecules-1399774-supplementary.pdf]

The semi-quantitative data of the chemical composition of EOs were subjected to one-way ANOVA to highlight significant differences ($p<0.05$). The analysis was performed within the farms (Table S1) by considering all the 12 EOs belonging to each farm, disregarding the type of treatment.

Table S1. One-way ANOVA results of significant differences between farms. The mean and the standard deviation (SD) of each terpene in each farm are reported and distinct letters statistically differ according to Tukey's post-hoc test ($p<0.05$). Homogeneous subsets are indicated by the same letter.

Terpene	(p<)	Farm	Mean ± SD (n=12)	*
α-pinene	0.0001	CA	0.55 ± 0.06	b
		PE	0.44 ± 0.06	a
		PR	0.57 ± 0.08	b
Camphene	0.0001	CA	0.47 ± 0.05	b
		PE	0.33 ± 0.03	a
		PR	0.36 ± 0.03	a
Sabinene	0.0001	CA	0.16 ± 0.03	b
		PE	0.13 ± 0.01	a
		PR	0.19 ± 0.03	c
β-pinene	0.0001	CA	0.15 ± 0.02	a
		PE	0.43 ± 0.06	b
		PR	0.61 ± 0.11	c
Oct-1-en-3-ol	0.0001	CA	0.92 ± 0.09	c
		PE	0.26 ± 0.04	b
		PR	0.19 ± 0.07	a
α-phellandrene	0.0001	CA	0.13 ± 0.03	b
		PE	0.07 ± 0.01	a
		PR	0.08 ± 0.01	a
3-carene	0.0001	CA	0.26 ± 0.06	c
		PE	0.1 ± 0.02	a
		PR	0.17 ± 0.03	b
Limonene	0.0001	CA	5.16 ± 2.01	b
		PE	0.91 ± 0.13	a
		PR	0.86 ± 0.27	a
1,8-cineole	0.0001	CA	3.21 ± 0.9	a
		PE	4.54 ± 0.41	b
		PR	5.85 ± 0.86	c
cis-ocimene	0.0001	CA	4.22 ± 0.71	b
		PE	1.29 ± 0.18	a
		PR	1.53 ± 0.15	a
trans-ocimene	0.0001	CA	1.64 ± 0.97	b
		PE	0.65 ± 0.16	a
		PR	0.63 ± 0.11	a
γ-terpinene	0.019	CA	0.35 ± 0.36	b
		PE	0.13 ± 0.02	a
		PR	0.13 ± 0.02	a

trans-Sabinene hydrate	0.013	CA	0.16 ± 0.08	b
		PE	0.09 ± 0.02	a
		PR	0.14 ± 0.04	b
cis linalool oxide	0.003	CA	0.13 ± 0.01	a
		PE	0.15 ± 0.01	b
		PR	0.14 ± 0.02	ab
trans linalool oxide	0.0001	CA	0.52 ± 0.06	b
		PE	0.46 ± 0.05	a
		PR	0.44 ± 0.03	a
Linalool	0.0001	CA	48.31 ± 3.71	c
		PE	33.4 ± 1.03	b
		PR	29.4 ± 1.54	a
Camphor	0.0001	CA	2.02 ± 0.37	a
		PE	6.42 ± 0.34	b
		PR	7.44 ± 0.34	c
trans-verbenol	0.0001	CA	0.16 ± 0.02	b
		PE	0.1 ± 0.01	a
		PR	0.1 ± 0.01	a
Borneol	0.0001	CA	11.39 ± 1.14	c
		PE	3.97 ± 0.32	b
		PR	2.65 ± 0.24	a
Lavandulol	0.0001	CA	0.64 ± 0.09	c
		PE	0.47 ± 0.04	b
		PR	0.41 ± 0.05	a
Terpinen-4-ol	0.0001	CA	5.38 ± 0.5	b
		PE	1.96 ± 0.08	a
		PR	2 ± 0.09	a
α-terpineol	0.0001	CA	0.27 ± 0.04	a
		PE	0.7 ± 0.12	b
		PR	0.73 ± 0.11	b
Myrtenal	0.0001	CA	0.51 ± 0.03	c
		PE	0.25 ± 0.01	b
		PR	0.2 ± 0.01	a
Nerol	0.0001	CA	0.16 ± 0.01	b
		PE	0.08 ± 0.02	a
		PR	0.08 ± 0.02	a
Carvone	0.0001	CA	0.14 ± 0.01	c
		PE	0.13 ± 0.01	b
		PR	0.11 ± 0.00	a
Lynalil acetate	0.0001	CA	4.88 ± 0.59	a
		PE	30.89 ± 1.69	b
		PR	32.66 ± 1.47	c
Lavandulyl acetate	0.0001	CA	1.2 ± 0.14	a
		PE	2.91 ± 0.11	b
		PR	2.89 ± 0.24	b
Neryl acetate	0.0001	CA	0.06 ± 0.01	a
		PE	0.27 ± 0.05	b
		PR	0.27 ± 0.05	b
β-cubebene	0.0001	CA	0.08 ± 0.02	a

		PE	0.58 ± 0.09	b
		PR	0.58 ± 0.09	b
β-caryophyllene	0.0001	CA	0.45 ± 0.05	a
		PE	1.55 ± 0.11	b
		PR	1.8 ± 0.14	c
α-bergamotene	0.0001	CA	0.06 ± 0.01	a
		PE	0.12 ± 0.01	b
		PR	0.16 ± 0.02	c
β-farnesene	0.0001	CA	2.14 ± 0.27	b
		PE	1.18 ± 0.11	a
		PR	1.28 ± 0.11	a
Ar curcumene	0.0001	CA	0.18 ± 0.04	a
		PE	0.68 ± 0.07	b
		PR	0.88 ± 0.08	c
δ-cadinene	0.015	CA	0.47 ± 0.09	a
		PE	0.55 ± 0.07	b
		PR	0.57 ± 0.09	b

One-way ANOVA was also performed within the treatments in each farm. In table S2, the significant differences observed were reported.

Table S2. One-way ANOVA results of significant differences between treatments in farms. The mean and the standard deviation (SD) of each terpene in each treatment group in the same farm are reported and distinct letters statistically differ according to Tukey's post-hoc test ($p<0.05$). Homogeneous subsets are indicated by the same letter.

Preci					Campazzo				
	**p<**	**Treatment**	**Mean ± SD**	*****	**Terpene**	**p<**	**Treatment**	**Mean ± SD**	
Sabinene	0.012	CTRL	0.152 ± 0.024	a	neryl acetate	0.001	CTRL	0.07 ± 0.01	b
		CTRL W	0.214 ± 0.019	b			CTRL W	0.05 ± 0.01	a
		T1	0.189 ± 0.016	ab			T1	0.05 ± 0.01	a
		T2	0.212 ± 0.013	b			T2	0.05 ± 0.00	a
α-phellandrene	0.005	CTRL	0.065 ± 0.000	a	α-bergamotene	0.001	CTRL	0.06 ± 0.00	b
		CTRL W	0.077 ± 0.000	ab			CTRL W	0.05 ± 0.00	a
		T1	0.083 ± 0.008	b			T1	0.05 ± 0.00	a
		T2	0.082 ± 0.008	b			T2	0.07 ± 0.01	b
1,8-cineole	0.013	CTRL	4.75 ± 0.71	a					
		CTRL W	6.42 ± 0.44	b					
		T1	5.76 ± 0.30	ab					
		T2	6.48 ± 0.57	b					
Linalool	0.038	CTRL	31.05 ± 0.25	b					
		CTRL W	27.82 ± 1.24	a					
		T1	29.73 ± 1.33	ab					
		T2	28.99 ± 1.19	ab					
Camphor	0.003	CTRL	6.97 ± 0.14	a					
		CTRL W	7.67 ± 0.16	b					

		T1	7.40 ± 0.18	ab
		T2	7.70 ± 0.21	b
Borneol	0.001	CTRL	3.01 ± 0.02	b
		CTRL W	2.59 ± 0.16	a
		T1	2.55 ± 0.03	a
		T2	2.46 ± 0.24	a
Lavandulol	0.001	CTRL	0.48 ± 0.03	b
		CTRL W	0.38 ± 0.01	a
		T1	0.38 ± 0.01	a
		T2	0.37 ± 0.03	a
Terpinen-4-ol	0.032	CTRL	2.05 ± 0.05	ab
		CTRL W	2.00 ± 0.09	ab
		T1	2.07 ± 0.04	b
		T2	1.97 ± 0.05	a
Lavandulyl acetate	0.008	CTRL	3.11 ± 0.17	b
		CTRL W	3.06 ± 0.12	b
		T1	2.64 ± 0.04	a
		T2	2.75 ± 0.18	ab

Table S3. Weather conditions and soil parameter of the investigated farms. PE = Pedroni Paola, Zocca, Modena, Italy; PR = Preci Carlo, Villa d'Aiano, Castel d'Aiano, Bologna, Italy; CA = Campazzo, Montombraro of Zocca, Modena, Italy; a.s.l. = above sea level.

**Parameters**	**Farms**		
	**PE**	**PR**	**CA**
**Weather conditions during the year**			
Average Temperature (°C)	11	11	12
Total rainfall (mm)	630	665	620
Altitude a.s.l. (m)	758	805	727
Field exposure	E	S	N-W
**Soil**			
Clay (%)	40.95	26.22	42.15
Silt (%)	40.73	52.83	45.02
Sand (%)	18.32	20.95	12.83
Texture (USDA)	Silty Clay	Silt Loam	Silty Clay
pH (in water)	7.70	7.90	7.90
Organic matter (%)	3.21	1.45	2.39
Total nitrogen (%)	0.23	0.10	0.17
C/N	8.20	8.60	8.20
Exchangeable potassium (ppm)	262.50	218.13	367.50
Assimilable phosphorous (ppm)	8.43	7.01	10.85
